# Rough-Legged Buzzards, Arctic Foxes and Red Foxes in a Tundra Ecosystem without Rodents

**DOI:** 10.1371/journal.pone.0118740

**Published:** 2015-02-18

**Authors:** Ivan Pokrovsky, Dorothée Ehrich, Rolf A. Ims, Alexander V. Kondratyev, Helmut Kruckenberg, Olga Kulikova, Julia Mihnevich, Liya Pokrovskaya, Alexander Shienok

**Affiliations:** 1 Department of Migration and Immuno-ecology, Max Planck Institute for Ornithology, Am Obstberg 1, D-78315, Radolfzell, Germany; 2 Department of Arctic and Marine Biology, University of Tromsø, NO-9037, Tromsø, Norway; 3 Institute of Biological Problems of the North, Far-East Branch Russian Academy of Sciences, 685000, Portovaya str. 18, Magadan, Russia; 4 Institute for Waterbird and Wetlands Research IWWR e.V., Am Steigbügel 3, D-27283, Verden (Aller), Germany; 5 Faculty of Geography, Lomonosov Moscow State University, GSP-1, Leninskie Gory, RU-119991, Moscow, Russia; 6 Faculty of Biology, Lomonosov Moscow State University, GSP-1, Leninskie Gory, RU-119991, Moscow, Russia; Evolutionary Biology Centre (EBC), Uppsala University, SWEDEN

## Abstract

Small rodents with multi-annual population cycles strongly influence the dynamics of food webs, and in particular predator-prey interactions, across most of the tundra biome. Rodents are however absent from some arctic islands, and studies on performance of arctic predators under such circumstances may be very instructive since rodent cycles have been predicted to collapse in a warming Arctic. Here we document for the first time how three normally rodent-dependent predator species—rough-legged buzzard, arctic fox and red fox – perform in a low-arctic ecosystem with no rodents. During six years (in 2006-2008 and 2011-2013) we studied diet and breeding performance of these predators in the rodent-free Kolguev Island in Arctic Russia. The rough-legged buzzards, previously known to be a small rodent specialist, have only during the last two decades become established on Kolguev Island. The buzzards successfully breed on the island at stable low density, but with high productivity based on goslings and willow ptarmigan as their main prey – altogether representing a novel ecological situation for this species. Breeding density of arctic fox varied from year to year, but with stable productivity based on mainly geese as prey. The density dynamic of the arctic fox appeared to be correlated with the date of spring arrival of the geese. Red foxes breed regularly on the island but in very low numbers that appear to have been unchanged over a long period – a situation that resemble what has been recently documented from Arctic America. Our study suggests that the three predators found breeding on Kolguev Island possess capacities for shifting to changing circumstances in low-arctic ecosystem as long as other small - medium sized terrestrial herbivores are present in good numbers.

## INTRODUCTION

Food webs are a core structuring element of ecosystems. The trophic interactions within food webs are variable in space and time, and understanding this variability can provide important insights in ecosystem functioning [1]. Indeed a food web approach has been used to describe ecosystem variability within the arctic tundra biome [2, 3]. Central to this variability is which guild of herbivores is dominating in a given ecosystem as this may have both important bottom-up impacts on the predators and top-down impacts on the plant communities in the food web [4]. In most tundra ecosystems small mammal herbivores (i.e. small rodents like lemming and voles) are the dominant herbivore guild [4], and the dynamics of the tundra food web are typically ruled by strong multi-annual trophic interaction cycles driven by the cyclic dynamics of lemmings and voles [5]. Small rodents are therefore considered key species in the ecosystem. Terrestrial tundra ecosystems without small rodents are found on arctic islands such as Svalbard. While this high-arctic ecosystem, which harbors only few vertebrate species, is well studied [6, 7], little is known about trophic interactions in low arctic tundra in the absence of small rodents. At the same time, these interaction cycles now appear to be regionally fading out, possibly due to climate warming [8–11] and it is important to study tundra ecosystems without small rodents to investigate what may happen in the Arctic in the future. In this paper we describe the rare case of a low-arctic ecosystem without small rodents and focus on performance of three predators that regularly rely on small rodents as their main prey: rough-legged buzzard *(Buteo lagopus)* (hereafter buzzard), arctic fox *(Vulpes lagopus)* and red fox *(Vulpes vulpes)*.

The buzzard and the arctic fox are arctic predators with a circumpolar distribution. Their population density and breeding success track the changes in small rodent availability, thus showing cyclic dynamics [12–14]. However, both of these predator species are able to include other resources in their diet to compensate for low rodent abundance [15–18]. While it is clear that buzzards are able to breed in years of low density of rodents, we have so far no evidence that they are able to successfully breed in ecosystems where small rodents are lacking completely. The arctic fox, on the contrary, is known to be an opportunistic predator and occurs in many different food web contexts [18–20]. It can notably use exclusively marine resources (sea birds and remains of cetaceans and marine invertebrates) when living on the sea coast for instance in Svalbard or on Mednyi Island [7, 21], or shift to marine resources in years of low small rodent abundance [22]. In certain areas of the Arctic, arctic foxes are negatively affected by the expansion of red fox, a superior competitor and also a highly opportunistic feeder [23, 24]. In other areas, however, the two fox species seem to coexist with stable relative numbers [25].

**Fig 1:**
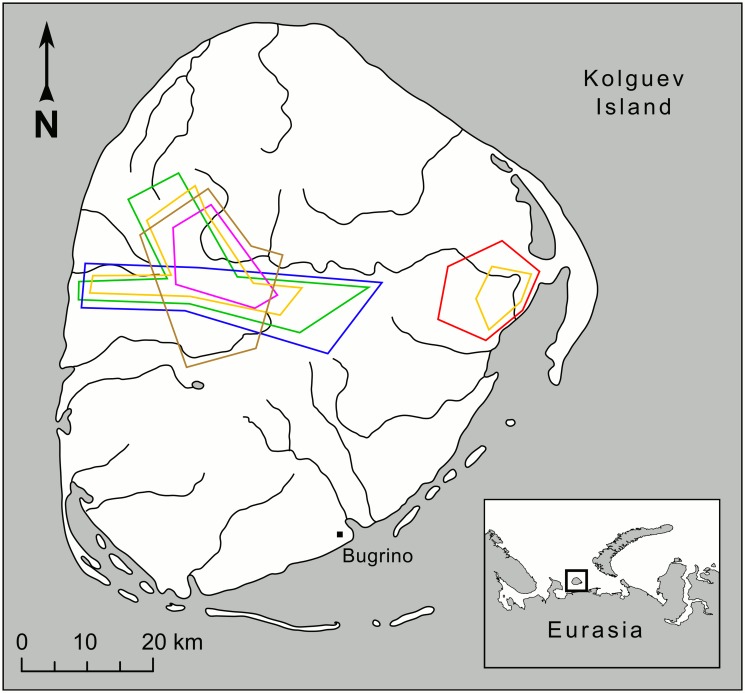
Map of Kolguev Island, Russia. Polygons show the approximate areas surveyed for buzzard nests and fox dens. Red polygon shows the survey area in 2006, green—in 2007, blue—in 2008, purple—in 2011, orange—in 2012 (two polygons), brown—in 2013.

On Kolguev Island in the Barents Sea the ecosystem is characterized by the total absence of all small to medium-sized mammalian herbivores; i.e. both rodents and hares. The area of the island is ca. 5000 km^2^, allowing for inland areas with relatively little marine influence (Fig. 1). The distance to the mainland is ca. 70–80 km. The primary vertebrate consumer guild consists mainly of birds. There is a large population of geese, including white-fronted goose (*Anser albifrons*), bean goose (*Anser fabalis*) and barnacle goose (*Branta leucopsis*). Moreover, the willow ptarmigan (*Lagopus lagopus*) is abundant. Semi-domestic reindeer (*Rangifer tarandus*) are the only mammalian herbivore. Insectivorous prey species include passerine birds and shore birds [26, 27]. Only reindeer are resident herbivores, as all birds, also most ptarmigans, leave the island for the winter. Morozov and Syroechkovsky [28] summarized investigations of birds on Kolguev Island and mentioned that from 1894 until 1928 buzzards were not listed among birds observed on the island in any of 23 publications from that period. After that, no ornithological investigations were carried out during 61 years (from 1928 until 1989). In 1989–1990, when a new survey was conducted, buzzards were not observed on Kolguev Island. In 1994–1995 two expeditions resulted in four observations of alarming buzzard pairs and three observations of single birds. The total effort consisted in two short visits in June and August 1994 including 534 km helicopter observation, and 25 days in June 1995. In 1997, Glazov found two nests (but did not note any information about content of the nests) and in 2003 one alarming pair and one single bird were registered [28–32]. Arctic foxes and red foxes were mentioned as a breeding species for Kolguev Island in 1894. The arctic fox was described as “far more abundant” than red fox [33]. In 1990–1997 arctic fox and red fox were also mentioned as a breeding species on Kolguev Island, however without any information about their relative density [31, 32, 34, 35]. Here we investigate the breeding performance and diet of rough-legged buzzards, arctic and red foxes on Kolguev Island. For the first time we report successful breeding of buzzards in complete absence of small rodents. The main objectives of our investigation were first to estimate the density and productivity of buzzards, arctic foxes and red foxes on the island in order to compare their breeding performance to that observed in other areas and to assess the relative breeding performance of the three species in absence of small rodents. Second, we aimed at determining the diet composition of buzzards and arctic foxes.

We predict that geese would constitute the main part of the predators’ diet in summer. As they are abundant and their population does not undergo important short-term fluctuations, it is likely that the productivity of the predators would be rather stable from year to year. Density of breeding pairs, on the contrary, may be determined by different factors for the migratory rough-legged buzzard and the more permanently resident foxes. Fox density could depend on the availability of resources in winter, such as reindeer carrion, as it was shown for Svalbard [6, 7, 36], whereas buzzard density may depend either on conditions in the wintering or migration areas or on local conditions at the time of arrival to the breeding grounds. Therefore we would not necessarily expect a correlation between the breeding densities of the species. The results are discussed in relation to climate-induced changes in tundra ecosystems. Altogether our intention is to contribute to a better understanding of the dependence of arctic predators with different degrees of diet specialization on the presence of small rodents, and thus the plasticity of food webs in tundra ecosystems.

## MATERIAL AND METHODS

### Ethics statement

No specific permissions were required for these location, species and activities according to the §44 and §6 of the Federal Law of the Russian Federation No. 52 from 24.04.1995 (last update 07.05.2013) “On Wildlife”. There were no Special Protected Natural Territories in our study area (described below in section “Study area”, including coordinates), our study species (rough-legged buzzard, arctic fox and red fox) were not listed in the Red List of Russian Federation, and our activities (described below in sections “Breeding population density and productivity parameters” and “Diet analysis”) did not include withdrawal of investigated species from nature.

### Study area

Our investigations were carried out on Kolguev Island in the Barents Sea (69°16’N 48°87’E), Russian Arctic, during six years: 2006–2008 and 2011–2013. The minimum and maximum temperatures on the island are -45°C and +30°C respectively; the average annual precipitation is 345 mm. The flora of the island is typical for the bio-climatic subzones E and D of the tundra biome (low arctic), and belongs to dwarf-shrub and low-shrub tundra types [37]. The valleys are characterized by poor meadow vegetation and dense shrub thickets ca. 0.5 m high. The thickets are composed of several species of willows *(Salix glauca*, *Salix lanata*, *Salix phylicifolia* and others) and dwarf birch *(Betula nana)*. The surrounding tundra consists of a rolling landscape with sand hills up to 150 m above sea level. Geese are the most abundant herbivore. The total estimated number of adult geese breeding on the island is 400,000–600,000 white-fronted geese, 170,000 barnacle geese and 30,000–60,000 bean geese [38]. Breeding parameters of the geese populations (density and nest initiation date) were obtained from the report of the expeditions to Kolguev by Institute for Waterbird and Wetlands Research [39]. The herd of semi-domestic reindeer comprises ca. 7,000 animals, which are not herded during summer and move freely around the island. Formerly there were two villages on the island, but now only one of them is inhabited (population is ca. 430). Availability of the reindeer carrion as potential resource for foxes in winter was evaluated based on the number of dead reindeers, which was inferred from the annual counts carried out by the herders in October-November each year.

Our study area was composed of two areas situated in the lower and middle course of the river Peschanka (Fig. 1). In 2006 the study was conducted in the lower course of the river, in 2012—in both locations and in other years—in the middle course of the river. This resulted in a study area of ca. 150 km^2^ in 2006 and 2011, ca. 250 km^2^ in 2012 and ca. 350 km^2^ in 2007, 2008 and 2013. We surveyed the area during walking excursions, using 8–10 X binoculars to search for raptors nests and fox dens. During these excursions we paid particular attention to river and lake banks, which are a common nesting habitat of buzzards.

### Breeding population density and productivity parameters

Density of rough-legged buzzards was estimated as the number of nesting pairs/100km^2^ in the monitored area in a given year. Nesting pairs were defined as pairs which had a specific territorial behaviour: female and male birds emitting “alarm calls” and circling over the intruder [40]. After detecting a nesting pair we started to search for the nest, moving to the direction where the volume and frequency of alarm calls were increasing. If the nest was not found after two hours, we stopped our search and registered a territory in the place where the alarm calls of the pair were most intensive. Coordinates of all nests and territories were determined using GPS navigators (Garmin, different models). The nesting success was assessed as the proportion of territorial pairs that raised at least one fledgling among pairs which had at least one egg [41]. Productivity of buzzards was estimated as the mean number of fledglings (young that reached 35 days of age—ca. 4 days before average fledging) in a particular year [41] including all nests which had at least one egg.

Density of arctic and red foxes was estimated as number of breeding pairs/100km^2^. Breeding pairs were defined as pairs that had at least one breeding den with pups (one or more). A den was considered as breeding if there were clear signs of the presence of pups on the den: visual observation of pups, and/or vocalization of pups from the den. The number of pups was determined during den observation on a day without rain in August. Productivity of foxes within the monitoring area was estimated as the mean number of pups per litter each year. In 2013 productivity of three arctic fox dens was evaluated with automatic cameras (Digital Ranger W50 RB with Sony Cyber-shot DSC-S700 camera; Camtrak South Inc.), taking one picture every five minutes (time lapse).

Fox species identity of occupied breeding dens was determined by visual observations of adults and/or pups. The two fox species can in some cases use the same dens, cf Gallant, Slough [25], however in most cases their dens can be distinguished by location in the landscape and the number of entrances. Non-breeding dens were thus attributed to one of the species according to the following criteria: dens with three or more entrances located on small hills were recorded as arctic fox dens, whereas dens with one or two entrances located in a river or lake bank—as a red fox den.

### Diet analysis

Using methods such as analyses of prey remains and scats or pellets for studying diet leads to certain biases in estimating prey proportions; however the combination of these methods allows to compensate for over- and underestimation of certain prey species in the diet [42–45]. In this study we combined data from these two methods. The buzzards’ diet was studied in 2013 and diet of arctic fox in 2012–2013 (in 2012 only prey remains were analyzed, but in 2013 prey remains analysis was combined with scat analysis). Prey remains were analyzed in the field and marked to avoid repeated counting. Prey remains found at the dens/nests were identified to species level or attributed to a group of species (e.g. unidentified goose or insect) and counted. We estimated age as fresh (belongs to the current year) and old (belongs to the previous years) for all prey remains found on fox dens in 2013. During visits to nests and dens, pellets and scats were collected for further analysis. A minimum number of prey items was determined for each species or group of prey in each batch of pellets/scats (i.e. all pellets/scats found at one place on one collection date). This estimation was based on the total number of skeletal parts identified in the batch. Thus, if one scull, four left legs and three right legs of a certain species or group were found in one batch, we concluded that this corresponded to at least four individuals. Uncountable remains (feathers, fur) were registered as one prey item for each batch of pellets where they were found, but only if there were no other remains of this species.

To quantify the diet of buzzards and foxes we first determined the list of prey species and the total number of prey items consumed by buzzards and foxes for each nest/den. To avoid double counting, the number of prey items for each nest/den was the maximum number of individuals of a particular species found in the pellets/scats and prey remains together (not the sum). Thus, if for one nest/den five individuals of a certain species or group were found in pellets/scats and three of them were found in prey remains, we concluded that this corresponds to at least five individuals. To summarize the diet, we calculated the percentage of each prey species (group) for each nest/den and then used the mean and CI of these percentages for comparison among categories of prey [46, 47]. In the final calculation we used only nests/dens for which the total number of registered prey items was more than ten. Proportions of prey in the diet were based on the number of prey items.

For buzzards’ diet we analysed a total of 119 pellets and 172 prey remains from 8 nests. The diet of arctic foxes was described on the basis of 229 prey remains from 18 dens in 2012 and 175 prey remains from 20 dens in 2013. In addition, 100 scats from five breeding dens (20 scats from each den) were analysed in 2013, as well as 529 old prey remains from 42 dens.

### Data analysis

All calculations were done using the statistical platform R 3.0.2 (R Development Core Team 2013). When analyzing differences of foxes’ and buzzards’ productivity between years and difference in dietary proportions, we used CI instead of significance tests [48, 49]. We presented the means and CIs for all estimates and considered differences with non-overlapping CIs as providing strong evidence for a biological difference (see Schenker and Gentleman [50]). Confidence intervals were calculated based on the binomial distribution for proportions (diet) and based on the Poisson distribution for productivity.

## RESULTS

### Density and productivity

**Fig 2:**
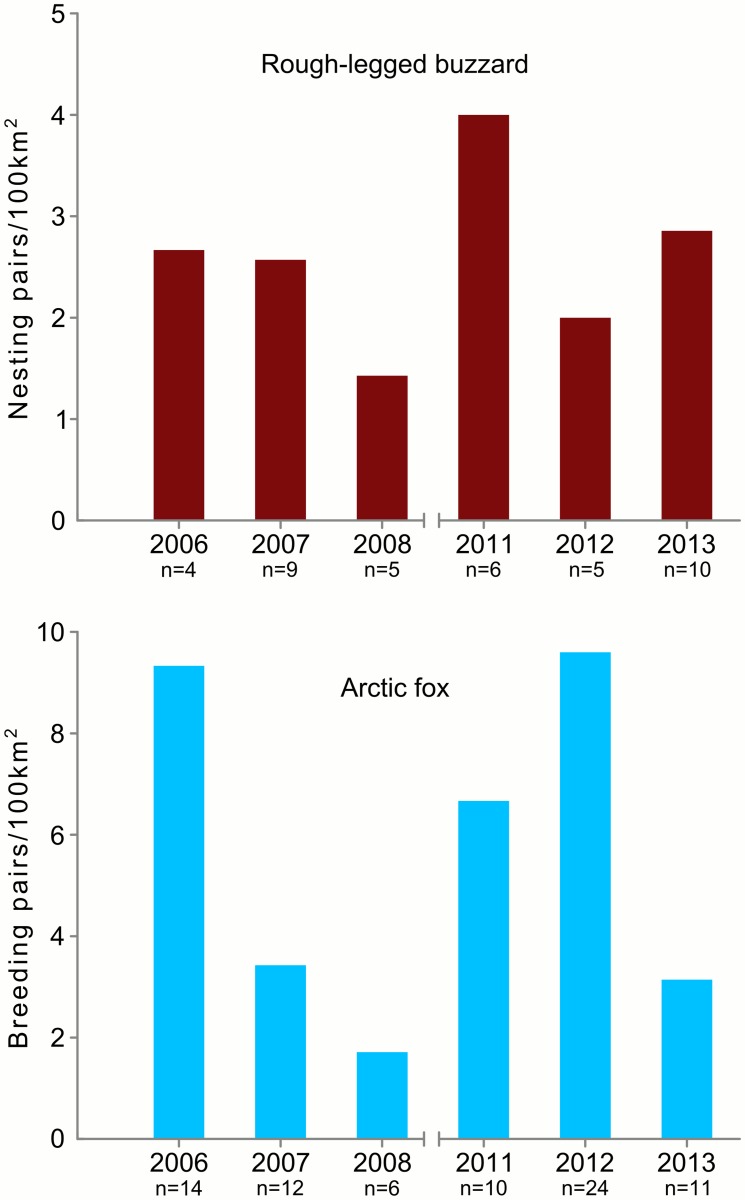
Density of rough-legged buzzards and arctic foxes on Kolguev Island, Russia. n—number of nesting pairs/breeding pairs documented in each year.

**Fig 3:**
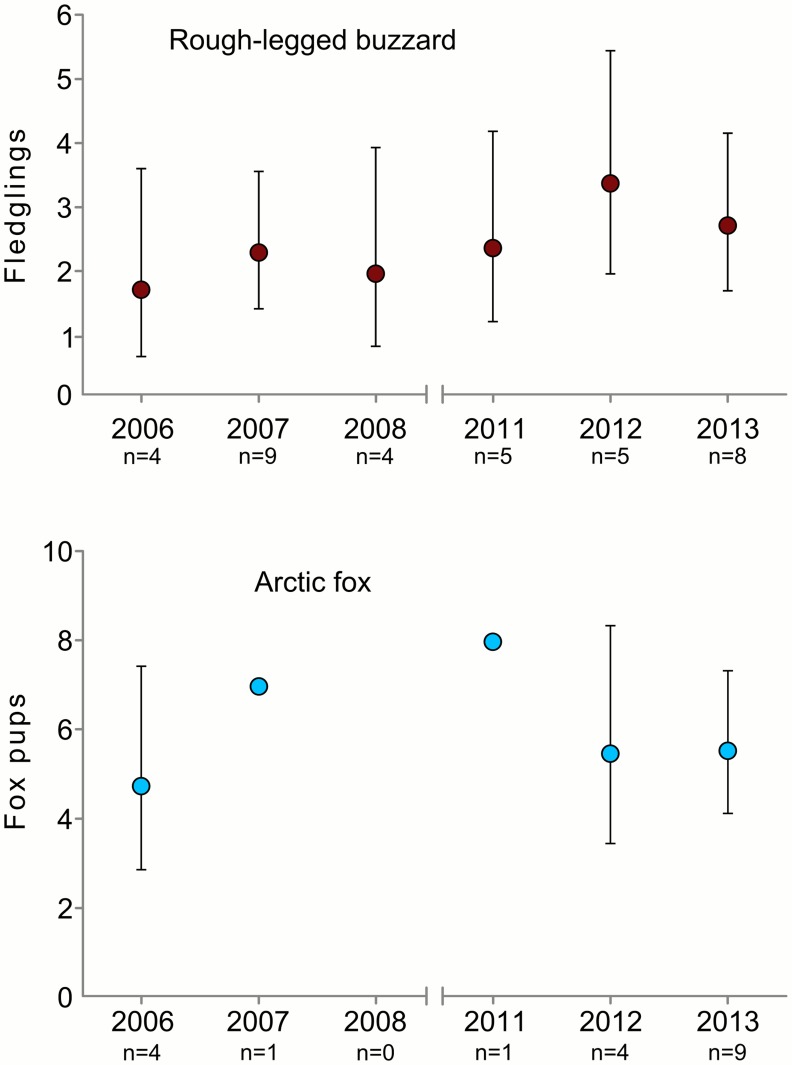
Productivity of rough-legged buzzards and arctic foxes on Kolguev Island, Russia. n—number of nests/dens with estimated productivity in each year. Error line—95% CI.

In total we found 39 nesting territories of buzzards and among them we found 35 nests. Two nests had no clutch; these nests were built on a slope covered by snow. When the snow melted the nests were destroyed by sliding down. Nestling mortality was documented in two nests: in one nest one out of five nestlings died and in another one all two nestlings died. Thus, the nesting success was 0.94±0.04 (mean ± SE) and survival rate of nestlings was 0.97±0.02 (mean ± SE). The density of buzzards varied from 1.4 to 4 nesting pairs/100 km^2^ (Fig. 2). Productivity of buzzards was estimated for all 35 nests and varied from 1.75 (95% CI = 0.7–3.61) to 3.4 (95% CI = 1.98–5.44) fledglings per nest in different years (Fig. 3). In 2006–2008 buzzards’ productivity was 2.11 (95% CI = 1.48–2.93) fledglings per nest and in 2011–2013 it was 2.83 (95% CI = 2.11–3.72).

We found 79 arctic fox dens in total; in 64 of them arctic fox bred at least in one of the years. Arctic fox density varied from 1.7 to 9.6 breeding pairs/100km^2^ (Fig. 2). In 2012 density of arctic foxes in the lower course of the river (50 km^2^) was 14 breeding pairs/100km^2^ and in the middle course of the river (200 km^2^)– 8.5 breeding pairs/100km^2^. Productivity of arctic fox was estimated for 19 dens in total, of which 17 were observed in the years 2006, 2012–2013. During this period productivity did not change and was on average 5.58 (95% CI = 4.57–6.75) pups/breeding pair (Fig. 3).

During all years we found 16 dens of red fox and in four of them red foxes bred at least in one year. Except 2012 we documented one breeding den of red foxes in each year. Thus, in 2006 and 2011 density of red foxes was 0.67 breeding pairs/100km^2^ and in 2007, 2008, 2013–0.29 breeding pairs/100km^2^. In 2007 and 2011 they bred in the same den. In 2011 red foxes had 8 pups and in 2013 we saw two pups, but obtaining reliable estimates of their productivity was not possible, because they move their pups to another den after the first visit at the den. In 2013 red foxes bred in an old arctic fox den.

**Fig 4:**
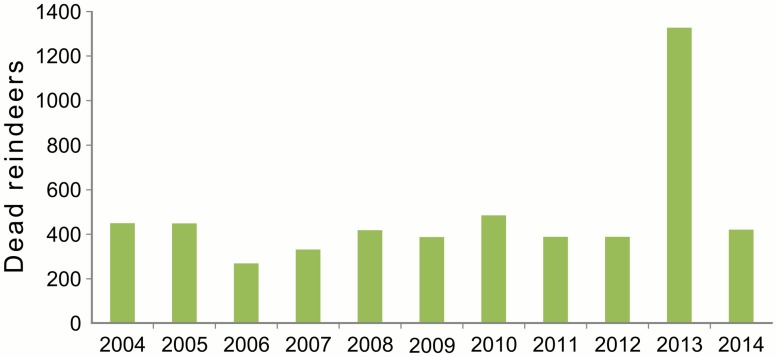
Number of the dead reindeers in 2004–2014 on Kolguev Island, Russia.

**Fig 5:**
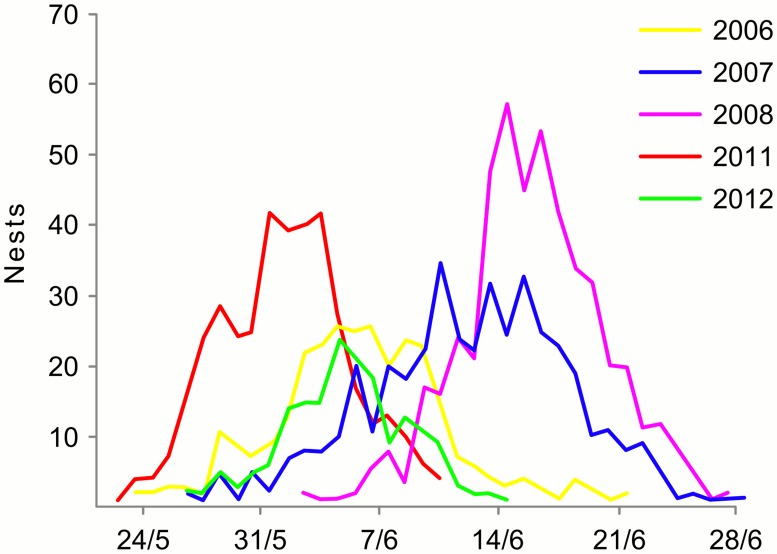
Nest initiation dates of white-fronted geese in 2006–2012 on Kolguev Island, Russia. From the report of the expeditions to Kolguev by Institute for Waterbird and Wetlands Research [39].

Number of the dead reindeers increased from 270 to 418 in 2006–2008; in 2010–2011 it was on the same level (ca. 400) and increased in 2013 to 1327 (Fig. 4). Density of geese was stable for white-fronted and bean geese and gradually increasing for barnacle goose [39]. The peak of nest initiation of white-fronted geese, which represent 2/3 of all geese population on the island, was earliest in 2006 and 2011–2012, whereas in 2007–2008 it was delayed by ca. 10 days (Fig. 5).

### Diet analysis

**Fig 6:**
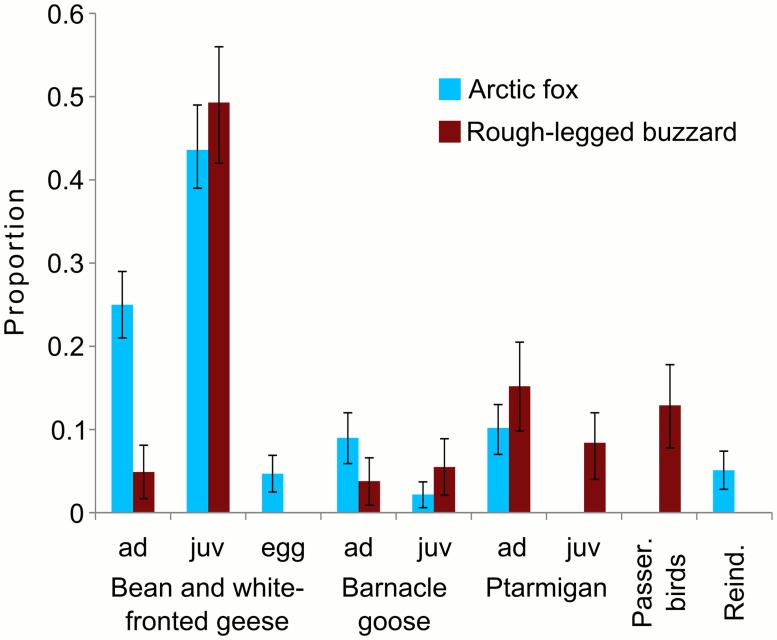
Diet of rough-legged buzzards and arctic foxes on Kolguev Island, Russia. Error line—95% CI. Passer. birds—passerine birds, Reind.—reindeers.

The diet of buzzards comprised nine species or groups of bird species (Table 1). Remains of a wader and of an arctic skua *(Stercorarius parasiticus)* were documented only once, and for one nest there were only seven prey items. These prey categories and this nest were not included into further analysis. Among the remaining 175 prey items from seven nests, geese represented 63% of all prey items, ptarmigans—24% and passerine birds—13% (Fig. 6).

**Table 1:** Number of identified prey items of Rough-legged buzzard and Arctic fox collected on the dens/nests on the Kolguev Island, Russia.

Species	Rough-legged buzzard	Arctic fox		
	2013	2012	2013	old
Bean and White-fronted geese (ad)	7	58	22	
Bean and White-fronted geese (juv)	107	93	105	
Bean and White-fronted geese (egg)		19	12	
Barnacle goose (ad)	9	14	17	
Barnacle goose (juv)	10	8		
Barnacle goose (egg)		3		
Goose sp. (ad)				450
Goose sp. (juv)				3
Swan sp.				1
Ptarmigan (ad)	23	19	12	62
Ptarmigan (juv)	8		1	
Ptarmigan (egg)		1	1	
Bluethroat	1			
Little Bunting	8			
Red-throated Pipit	2			
Lapland Bunting	3	1		
Passerine sp.	2			
Wader sp.	1			1
Dunlin (juv)		1		
Parasitic Skua	1			
Reindeer (juv)		12	4	3
Reindeer (ad)			1	9
Insects			21	
Berries			4	
*Number of nests/dens*	*8*	*18*	*20*	

The numbers represent the sum of prey remains and items identified during pellet/scat analysis.

The diet of arctic foxes consisted of six species of birds and one mammalian species (Table 1). In 2012 ten dens had more than ten fresh prey items and in 2013—five dens; on these dens we found a total of 145 and 199 prey remains respectively. Among these prey remains geese composed 84% of all prey items, ptarmigans—11% and reindeer—5% (Fig. 6). On 27 dens we found more than ten old food remains. Among 443 old food remains geese composed 86% of all prey items, ptarmigans—12%, reindeer—2%. In addition, we found single remains of a wader and a swan (Table 1).

## DISCUSSION

### Rough-legged buzzards

Here we report for the first time breeding of rough-legged buzzards in an area without small rodents. Buzzards nested on Kolguev Island for at least six years at relatively stable and low density (2.6±0.7 nesting pairs/100km^2^) and used birds (mostly geese) as prey to feed their nestlings. The productivity of buzzards on Kolguev Island was relatively stable among years and did not drop to zero in any of the six years of the study. This stability is in contrast to the dynamics of the species in most ecosystems with small rodents, where density and productivity of buzzards track the density of rodents. In ecosystems with high-amplitude small rodent cycles nesting buzzards are usually absent during rodent crash years [51, 52]. Yet in other ecosystems, where rodent cycles appear somewhat less strong and where alternative prey are abundant, buzzard breeding densities are more stable [15, 17]. The density of breeding buzzards observed on Kolguev is similar to what is observed in other areas at low small rodent densities [51–53]. However, in areas with high amplitude rodent cycles breeding densities during peak years can be up to 10 times higher [52]. Nesting success of buzzards on Kolguev was very high and the survival rate of the nestlings was one of the highest which has been reported for buzzards [17, 51–54]. Given the available historical data [28], it appears that buzzards have only recently started to breed regularly on Kolguev Island. While the species seemed absent from the island 100 years ago, single possible breeding events were registered, but not confirmed in the 1990s.

Buzzards are nomadic raptors and during spring migrations they choose a nesting area while observing territory for high density of rodents [13]. When rodent peaks are absent over large areas, buzzards could pass the breeding season. Buzzards established on Kolguev Island in the same period as a large-scale absence of high-amplitude cycles of rodents in Europe [55]. Thus, the appearance of buzzards on Kolguev Island is likely to be a consequence of the absence of their main food resource in the normal breeding range in northern boreal and Arctic Europe. This shows that the foraging behavior is more plastic than usually assumed for this species and thus an ability to adapt to new environmental conditions.

High nesting success and survival rate of nestlings indicate good breeding conditions for buzzards, however the density of buzzards is still relatively low and did not show any consistent trends of increase over the study period. The density of buzzards on Kolguev Island may also depend on the density of small rodents on the mainland around the island. Such a relationship could be suggested by the observation of high density of rodents on the Nenetsky Ridge in 2008 [17] and low density of buzzards in that year on Kolguev Island. Also cyclic peaks of rodents in the north-western Europe returned after 2007 [55] and, this may have slowed down the increase of number of nesting buzzards on Kolguev Island. Whether the population of buzzards on Kolguev Island is distinct from mainland populations needs to be established by other study approaches. In particular, future studies of migration pathways of adults and juvenile birds can be instructive. Currently, very little is known about migration pathways of buzzards.

Among the three species of geese, buzzards consumed mostly juveniles of bean and white-fronted geese; barnacle geese were not so often consumed by predators, at least in the central part of the island. In this part of the island the density of barnacle geese is ca. 3 times lower than density of bean and white-fronted geese, while the proportion of barnacle geese in the diet of buzzards and arctic foxes was ca. 6 times lower. This could be a result of different ways of brood protection by adult geese. In proximity of both avian and mammalian predators families of barnacle geese usually gather in dense flocks protected by several adults, while families of both bean and white-fronted geese usually run and try to hide in thickets of shrubs separately, often loosing goslings during these fast escapes.

Altogether, we showed that buzzards may be able to adapt to new environmental conditions and to a tundra ecosystem without small rodents. Buzzards can completely shift from rodent prey to ptarmigans and geese and are able to successfully reproduce using them as a main food resource.

### Arctic fox and Red fox

Breeding density of arctic fox varied during our study in an irregular way. The productivity of arctic foxes was not estimated properly in all years, however in 2006, 2012 and 2013 it was approximately on the same level with a litter size that resemble other population of arctic foxes without access to rodents [56]. Some of the between-year variation in population density could be explained by different sizes and location of the survey area. Thus, in 2006 and 2012 the study area included the lower reaches of the river Peschanka, where a big colony of barnacle geese is situated. That could explain the high density of arctic fox observed in these years. However, the density of arctic foxes differed also among the other years, when the same area was surveyed. Food resources in this area, which consists mainly from the white-fronted and bean geese, in summer, were stable and abundant in all years and this led us to conclude that density dynamic of arctic fox population could be driven by other factors than density of geese in the breeding season. A likely candidate factor is goose breeding-phenology, which exhibits between-year variability on Kolguev Island (Fig. 5). For successful reproduction arctic fox breeding should be synchronized with the breeding of geese, which constitute their main food resource. Indeed, arctic fox breeding density co-varied with the date of geese nest initiation; i.e. more foxes were breeding in the years when geese arrived earlier. Arctic foxes mate in February, when it is impossible to predict the date of geese arrivals. In the years with late spring, and thus late arrival of geese to the island, it could lead to mismatched breeding phenology of the arctic to phenology of their main prey. Thus, the main factor controlling the density of arctic foxes could be the time of goose nest initiation. Date of geese arriving dependent from the weather conditions and in warming climate it could be assumed to take place earlier [57], which could positively affect population of arctic foxes on Kolguev Island.

In high-arctic Svalbard availability of the reindeer carrion as determined by winter climate variability is the strongest predictor of the breeding population of arctic fox [6, 7, 36]. However, contrary to our expectations, the year-to-year variation in density of breeding arctic foxes on the low-arctic Kolguev Island showed no obvious relationship with the annual reindeer mortality on the island. We can only speculate about the reason for this. One reason could be inaccuracies in the number of dead reindeer estimated by the herders. Another reason could be that the amount of reindeer carrion was always above the threshold for high fox survival during winter. Finally, a large proportion of arctic foxes may migrate from the island during winter, similar to what has recently been documented on the Bylot Island in the Canadian Arctic [58].

The presence of semi-domestic reindeer could be essential for the presence of red fox on Kolguev [59]. However, red foxes are still quite rare on Kolguev as they were more than hundred years ago. Although red foxes are expanding northwards in certain areas in the Arctic [23] and seem to have the potential to outcompete the arctic fox in northern Scandinavia [60], in other areas the two species seem to co-exist in stable numbers [25]. Notably on Bylot Island the relationship between the two species is similar to what we observed on Kolguev, without major changes in recent years. The results of Gallant et al. [25] indicate that the intensity of climate change in itself does not explain these differences between regions. A limiting factor for all resident predators in the Arctic is food availability during winter. Red foxes are larger than arctic foxes and less tolerant to cold and starvation [18]. Consequently they have higher energy demands during winter making them dependent on resource subsidies [59]. Compared to arctic foxes red foxes are probably also less nomadic during winter contributing to their higher dependence on year-round stability of food resources in the breeding areas. With its very rich and temporally stable resources during the summer season, but absence of such resource richness and stability in the winter, the ecosystem in Kolguev seems to suit arctic foxes better than red foxes.

### Guild of predators

Geese constitute the main food resource for both buzzards and arctic foxes and indeed the entire terrestrial predator community of Kolguev Island appear to depend on geese. Still temporal variation in breeding density may not be synchronized between species in the same manner species within the guild of arctic rodent predator typically are. For example, compared to arctic foxes buzzards seems not to be able to rely on adult geese as prey and compensate this by preying on goslings, ptarmigan and passerine birds. Further as discussed above, the density of buzzards on Kolguev may depend on the dynamics of rodent on the mainland close to the island. Moreover, arctic foxes (initiating breeding very early in the year) should be most sensitive to goose breeding phenology. Finally, red foxes are more vulnerable to cold winters and starvation than arctic foxes.

### Implications and perspectives

Climate warming is particularly rapid in the Arctic and already affecting terrestrial tundra ecosystems [3, 24, 61]. One key component of life in the tundra is the lemming cycle that provides abundant resources for numerous predators every three to five years. This heartbeat of the arctic ecosystem is however sensitive to changes in winter climate and has faded out during the last decades in several regions [8–11] with possibly dramatic consequences for predators [18, 62]. At the same time goose populations have increased dramatically in certain areas of the arctic [63, 64]. Investigating the ability of arctic predators to switch from rodents to other resources will be a key moment for the understanding of their future fate. Here we have shown that two of the most common arctic predators across the tundra biome have certain adaptive capacities for performing such switches.
